# BasePhasing: a highly efficient approach for preimplantation genetic haplotyping in clinical application of balanced translocation carriers

**DOI:** 10.1186/s12920-019-0495-6

**Published:** 2019-03-18

**Authors:** Shuo Zhang, Dingding Zhao, Jun Zhang, Yan Mao, Lingyin Kong, Yueping Zhang, Bo Liang, Xiaoxi Sun, Congjian Xu

**Affiliations:** 1 Shanghai Ji Ai Genetics & IVF Institute, Obstetrics and Gynecology Hospital, Fudan University, Shanghai, 200011 China; 2 Key Laboratory of Female Reproductive Endocrine Related Diseases, Obstetrics and Gynecology Hospital, Fudan University, Shanghai, 200011 China; 3 Basecare Medical Device Co., Ltd, 218 Xinghu Road, SIP, Suzhou, Jiangsu 215001 China; 4 State Key Laboratory of Microbial Metabolism, Joint International Research Laboratory of Metabolic and Developmental Sciences, School of Life Sciences and Biotechnology, Shanghai Jiao Tong University , 800 Dongchuan Road, Shanghai, 200240 China; 5 State Key Laboratory of Genetic Engineering, Collaborative Innovation Center for Genetics and Development, School of Life Science, Fudan University, 588 Fangxie Rd, Shanghai, 200438 China

**Keywords:** Preimplantation genetic testing, Balanced translocation, Infinium Asian screening Array-24, Preimplantation genetic haplotyping, BasePhasing

## Abstract

**Background:**

Preimplantation genetic testing (PGT) has already been applied in chromosomally balanced translocation carriers to improve the clinical outcome of assisted reproduction. However, traditional methods could not further distinguish embryos carrying a translocation from those with a normal karyotype prior to implantation.

**Methods:**

To solve this problem, we developed a method named “Chromosomal Phasing on Base level” (BasePhasing), which based on Infinium Asian Screening Array-24 v1.0 (ASA) and a specially phasing pipeline. Firstly, by comparing the number of single nucleotide polymorphism (SNP) loci in different minor allele frequencies (MAFs) and in 2Mbp continuous windows of ASA chip and karyomap-12 chip, we verified whether ASA could be adopted for genome-wide haplotype linkage analysis. Besides, the whole gene amplification (WGA) of 3–10 cells of GM16457 cell line was used to verify whether ASA chip could be used for testing of WGA products. Finally, two balanced translocation families were utilized to carry out BasePhasing and to validate the feasibility of its clinical application.

**Results:**

The average number of SNP loci in each window of ASA (473.2) was twice of that of Karyomap-12 (201.2). The coincidence rate of SNP loci in genomic DNA and WGA products was about 97%. The 5.3Mbp deletion was detected positively in cell line GM16457 of both genomic DNA and WGA products, and haplotype linkage analysis was performed in genome wide successfully. In the two balanced translocation families, 18 blastocysts were analyzed, in which 8 were unbalanced and the other 10 were balanced or normal chromosomes. Two embryos were transferred back to the patients successfully, and prenatal cytogenetic analysis of amniotic fluid was performed in the second trimester. The results predicted by BasePhasing and prenatal diagnosis were totally consistent.

**Conclusions:**

Infinium ASA bead chip based BasePhasing pipeline shows good performance in balanced translocation carrier testing. With the characteristics of simple operation procedure and accurate results, we demonstrate that BasePhasing is one of the most suitable methods to distinguish between balanced and structurally normal chromosome embryos from translocation carriers in PGT at present.

**Electronic supplementary material:**

The online version of this article (10.1186/s12920-019-0495-6) contains supplementary material, which is available to authorized users.

## Background

Balanced translocation is one of the most frequent indications for preimplantation genetic testing (PGT), which occurs at an incidence of 1/500 to 1/625 in the general population and even up to 1/20 in the patients who has a history of repeated IVF failure or recurrent miscarriages [1]. Although they often have normal phenotypes, but the risk of producing unbalanced gametes is high (typically approximately 70%) due to the abnormal segregation of rearranged chromosomes during meiosis [2, 3]. The unbalanced gametes will lead to apparent infertilities [4], recurrent miscarriages [5, 6] or other congenital abnormalities [4, 7]. Therefore, it is of great significance to prevent balanced translocations from being passed to the next generation by assisted reproductive technology (ART) and PGT.

The first PGT case for translocations reported in 1998 was used by fluorescence in situ hybridization (FISH) [8]. However, the application of FISH is limited by some technical problems, such as ambiguous signals and complex operation in detecting limited chromosomes [9–11]. With the development of technology, aCGH/SNP array [12, 13] and whole genome sequencing [14, 15] methods have been employed for balanced translocation detection. Although these traditional PGT methods can clearly identify embryos with chromosomally unbalanced translocation or aneuploidies, they can hardly further distinguish the balanced and structurally normal embryos. Over the past 5 years, researchers tried to use mate pair sequencing [16, 17], MicroSeq-PGD [18], MaReCs [19], and long read sequencing [20] to obtain the precise breakpoints of balanced translocation, followed by subsequent identification of normal embryos through PCR-Sanger sequencing or linkage analysis based on the breakpoints. However, it remains unstable and inaccurate to identify the precise breakpoints in the highly repetitive and variable translocation regions.

In our former study [21], we successfully utilized preimplantation genetic haplotyping (PGH) to distinguish balanced and structurally normal embryos prior to implantation for both reciprocal translocation and Robertsonian translocation carriers accurately, along with the genetic screening for all 23-pairs of chromosomes. However, the used SNP microarray contained relatively less SNP loci. In another study [22], the researchers chose Human CytoSNP-12 BeadChip, which also faced the same trouble. Therefore, it remains a challenge to obtain a highly efficient approach to distinguish the normal embryos from those with a balanced translocation karyotype in clinical. In this study, based on our previous PGH technology theory, here we established a new method named “Chromosomal Phasing on Base level” (BasePhasing). More SNP loci (700 K SNPs) bead chip (Illumina Infinium Asian Screening Array-24 v1.0, ASA) was used to perform BasePhasing and a new analysis pipeline was developed for ASA data. BasePhasing was validated by the whole genome amplification result of cell line GM16457, and two balanced translocation families were also analyzed. The accuracy of this method was validated by the conventional amniotic fluid karyotypes in the second trimester.

## Methods

### ASA assessment for BasePhasing

The basic Microarray technical data of ASA and Karyomap-12 was downloaded from Illumina official website (https://www.illumina.com). As we known, the number of informative SNPs is more valuable to linkage analysis, which allows for directly determining the accuracy of haplotype classification. For a SNP to be informative, one parent must have a heterozygous genotype and the other one should have a homozygous genotype. This is limited by specific families and may also be affected by the distribution of SNP frequency. For the same family, SNPs with high MAF are more likely to generate the informative SNP.

Usually, the region within ±2Mbp flanking region of the breakpoints was chosen to avoid misinterpretation from possible recombination events that might occur during meiosis. More importantly, sufficient number of informative SNPs could be obtained in the 2Mbp region to distinguish homologous recombination. Therefore, the whole human genome could be divided into large amounts of 2Mbp segments (called windows). By comparing the number of total SNPs and effective SNPs in each widow, we can get analytical performance of ASA bead chip.

### Ethics statement

Written informed consent was obtained from each family and the study protocol was approved by the Ethics Committee for Human Subject research of the Obstetrics and Gynecology Hospital, Fudan University.

### Samples preparation and DNA isolation

Cell line GM16457 was obtained from the NIGMS Human Genetic Cell Repository at the Coriell Institute for Medical Research, with karyotype 46,XX,del(18)(q22.3) and a known 5.3Mbp deletion in chr18. Two translocation carrier families that would undergo assisted reproductive were enrolled in Shanghai Ji Ai Genetics & IVF Institute in May 2018. Both families had a history of recurrent spontaneous abortion, infertility or pregnancies with chromosome anomalies. The translocation karyotypes were 46,XY,t(1:5)(q21;q35) and 46,XY,t(6;7)(q23;q34) respectively. Five milliliter peripheral blood from each couple and family members was collected at recruitment.

For cell line GM16457 and peripheral blood samples, the high molecular weight DNA was isolated as described in the manufacturers’ protocol (DNeasy Blood & Tissue Kit, QIAGEN, Germany).

### Single cell preparation and WGA

Three to ten cells of the GM16457 cell line were isolated by micromanipulating under a dissection microscope (Olympus CKX41, Japan) using a finely pulled glass Pasteur pipette. For embryos at the blastocyst stage, three to ten cells were removed from the trophectoderm on day five of embryonic development. The biopsied cells were placed into 0 .2mL PCR tubes in a total volume of less than 4 .0μL. Whole genome amplification (WGA) was conducted by means of multiple displacement amplification (MDA) according to the manufacturer’s instructions (Repli-g single cell kit, QIAGEN, Germany). The isothermal amplification was carried out at 30 °C for 8 h and followed by enzyme inactivation at 65 °C for 3 min.

### SNP-array and analysis

The isolated DNA and WGA products were treated according to the manufacturer’s instructions (Illumina, San Diego, CA, USA), which were then scanned using an Illumina iScan Bead Array Reader. The microarray scanning results were processed using the B allele frequency and Log R Ratio of Genome Studio software (Illumina) and Karyo Studio software (Illumina) to analyze the copy number of the chromosomes. Genome wide preimplantation genetic haplotyping (PGH) analysis based on Illumina Human Karyomap-12 V1.0 microarray was performed as our previous description [21].

### BasePhasing

**Fig. 1:**
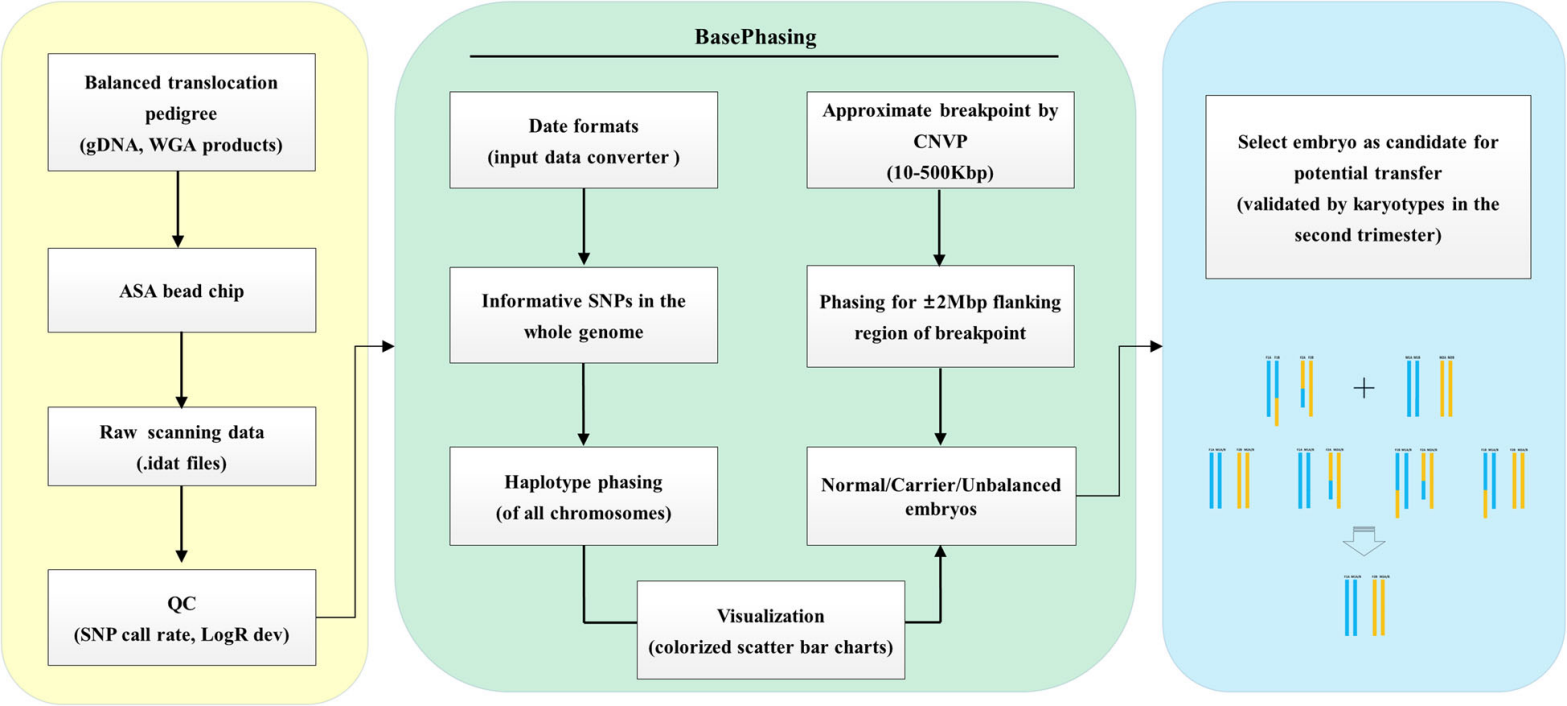
Overview of the BasePhasing pipeline. Summary of the most important features of the BasePhasing pipeline. Black arrows show the flow of information from the samples to the final identification results. ASA bead chip scanning data and quality control (yellow background), BasePhasing analysis (green background), and candidate embryos transfer (blue background). CNVP, CNV partition analysis algorithm

Based on informative SNPs and chromosomal phasing principles we developed BasePhasing pipeline, which was programmed in Practical Extraction and Reporting Language (Perl), and was capable of obtaining the clear haplotypes of each family member in linkage analysis (Fig. 1). The raw scanning data would be imported into the BasePhasing to produce the accurate chromosomal aneuploidy and haplotype results, once all the family samples were detected by ASA bead chip in a single test. To avoid misinterpretation precious statement, the region within ±2Mbp flanking region of the breakpoints was chosen to analyze the balanced translocation carriers.

Additionally, BasePhasing was a universal pipeline with compatibility for other bead chips such as Human Karyomap-12, Infinium Global Screening Array-24 (GSA), Human CytoSNP-12, and Infinium Omini series microarrays.

## Results

### ASA basic performance for BasePhasing

**Fig. 2:**
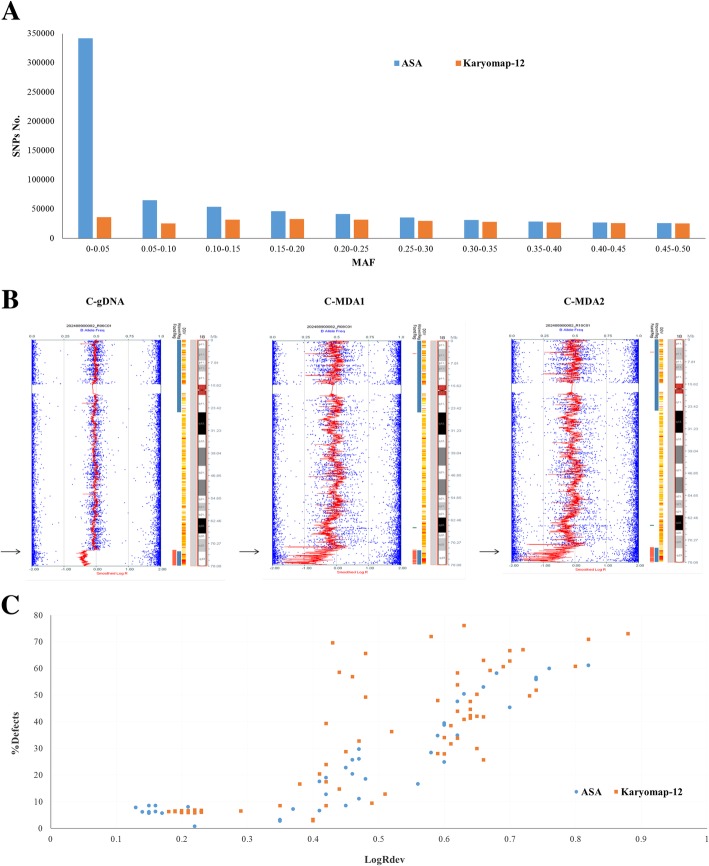
The performance of ASA bead chip in terms of SNP and CNV analysis. **a** SNPs distribution of ASA and Karyomap-12 bead chips. MAF: minor allele frequency. **b** A 5.3Mbp deletion in cell line gDNA and its single cell MDA products. Black arrows indicate the 5.3Mbp deletion in chr18. B allele frequency (BAF, genotyping information) represented by blue dots. Smoothed log R ratio represented by a red line. In addition, there is an ideogram of the chromosome, found and known regions, information from the DGV (Database of Genomic Variants), and gene information. **c** 136 ASA and Karyomap-12 bead chip data on the CNV performance for CCS. % Defect: Score given to each sample based on the number of detected regions. This value is the sum of the length of all detected regions per sample divided by the length of the genome. LogRDev: Standard deviation of the log R ratios of the sample. Sample type: 52 genome DNA and 84 MDA products

ASA bead chip contained approximately 700 K SNPs, more than double what those for Karyomap-12 bead chips. Although half amount of ASA SNPs MAF was lower than 0.1, the total number of high frequency SNPs was more than Karyomap-12 (Additional file 1: Table S1, Fig. 2a). Such as in MAF 0.25–0.5, ASA had total SNPs number 149,462 and Karyomap-12 was 136,690. In sum, ASA entirely had more SNPs than Karyomap-12 at every MAF segment.

The total number of windows in the whole genome was 1561, but some regions like centromeres and satellites had no SNP probes distribution, so the effective number of windows was 1476. The number of uniquely mapped SNPs in each window was determined (Table 1). To get enough number of informative SNPs for haplotype analysis, knowing that in typical cases approximately only 10–20% of SNPs were informative SNPs, we considered there should be at least 50 SNPs in each window. In the whole genome, ASA with 98.85% region met the requirements, and the average number of SNPs in each window was up to 473.2.

**Table 1:** Comparison of the SNPs number between ASA and Karyomap-12 in each window at different minor allele frequencies

Content	MAF = 0–0.5		MAF = 0.1–0.5		MAF = 0.25–0.5	
	ASA	Karyomap-12	ASA	Karyomap-12	ASA	Karyomap-12
SNPs No.	698,500	294,602	291,178	227,939	149,323	133,180
effective windows No.^a^	1476	1464	1461	1449	1458	1444
Average No. of SNPs in each window	473.2	201.2	199.3	157.3	102.4	92.2
≥50 SNPs windows No.	1459	1417	1395	1387	1257	1260
Accordance rate^b^	98.85%	96.00%	94.51%	93.97%	85.16%	85.37%

MAF: minor allele frequency

^a^the number of windows that had SNP loci in bead chip

^b^meaning the percentage of ≥50 SNPs windows number divided by the effective windows No

### Evaluation of BasePhasing feasibility

To assess ASA performance with single cell MDA products, we used both gDNA and single cell MDA products of cell line GM16457. Cell line GM16457 gDNA gave call rate of 98.7% and heterozygous call rate of 16.1%. SNP call rates were a little lower in single cells, about 96.3% and heterozygous call rates 14.8% (Table 2). We also compared the SNPs accordance between cell line gDNA and its single cell MDA products. It showed that the accordance rate was up to ~ 97%, not only between gDNA and its MDA products but also between the MDA products themselves. In addition, the known 5.3Mbp deletion in cell line GM16457 was clearly detected in MDA products (Fig. 2b).

**Table 2:** Cell line gDNA and its single cell MDA products tested by ASA bead chip

Sample ID	Call rate	% Defects^a^	LogRDev^b^	Heterozygous call rate	SNPs accordance vs C-gDNA
C-gDNA	0.9871739	1.56	0.15	16.1%	100%
C-MDA1	0.9642419	7.74	0.38	14.8%	97.01%
C-MDA2	0.9616035	7.76	0.39	14.8%	96.72%

Cell line: GM16457, with a known 5.3Mbp deletion in chr18; C-MDA1 and C-MDA2 are the repetition

^a^Score given to each sample based on the number of detected regions. This value is the sum of the length of all detected regions per sample divided by the length of the genome

^b^Standard deviation of the log R ratios of the sample

Except the SNPs amount and distribution, ASA could also perform well for comprehensive chromosome screening (CCS). The CCS analysis mainly relied on the SNP allele frequency analysis in the whole genome. We gathered and compared 136 previous samples data of bead chip in our laboratory, including 43 samples by ASA and 93 samples by Karyomap-12. We found that there were no differences between ASA and Karyomap-12 on the LogRdev versus %Defects (Fig. 2c), which signified the copy number variation (CNV) performance for CCS.

### BasePhasing clinical test

**Fig. 3:**
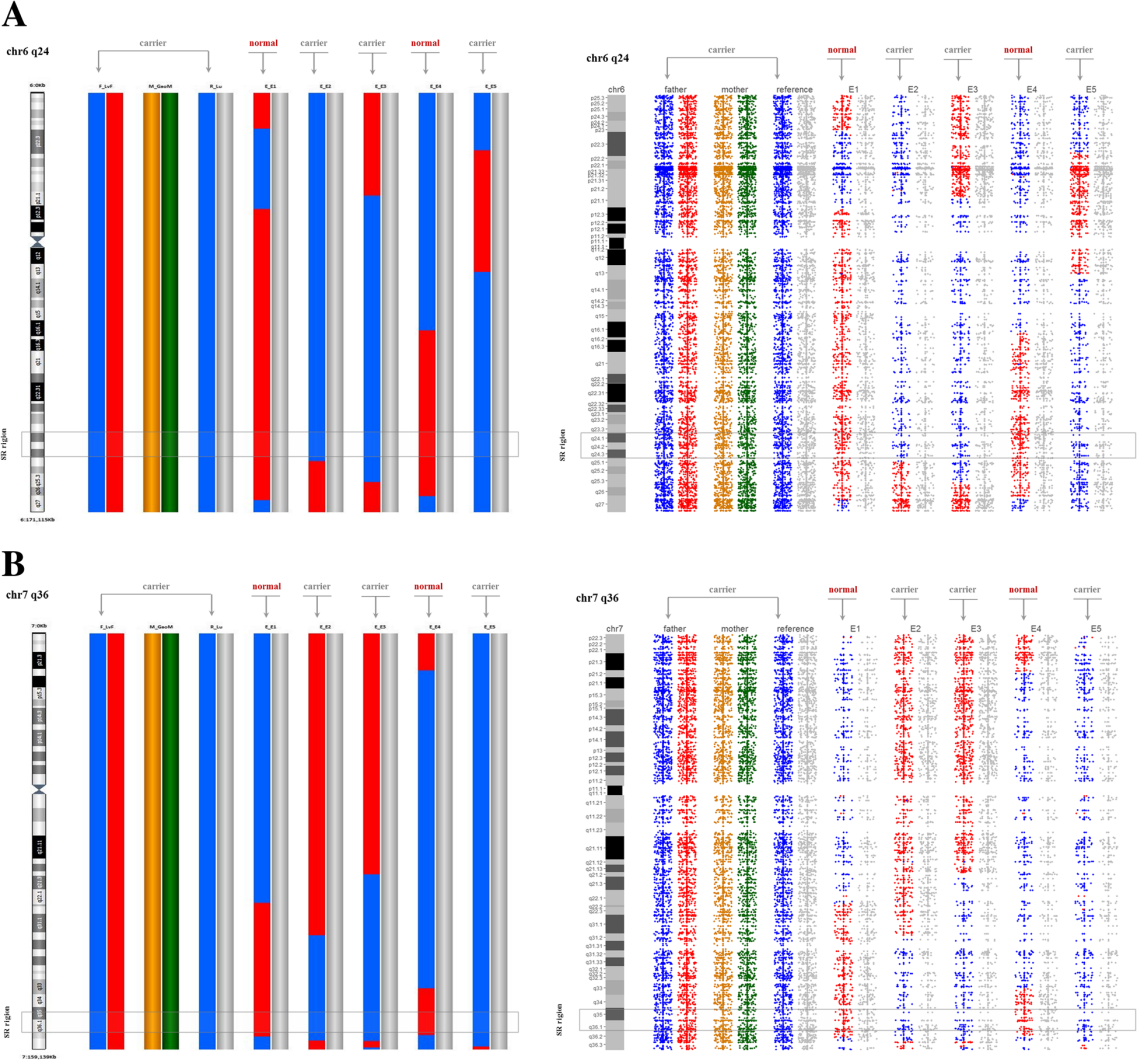
BasePhasing results of family 2 in the two balanced translocation breakpoints related chromosomes. **a** BasePhasing results of family 2 in the balanced translocation breakpoint related chromosome 6. The left smooth bar charts were performed with Blue-fuse-Multi software from Karyomap-12 bead chip data, the right scatter bar charts were performed with BasePhasing from ASA bead chip data. **b** BasePhasing results of family 2 in the balanced translocation breakpoint related chromosome 7. The left smooth bar charts were performed with Blue-fuse-Multi software from Karyomap-12 bead chip data, the right scatter bar charts were performed with BasePhasing from ASA bead chip data. SR region: the breakpoint of balanced translocation (gray box labeled). The different colorful histograms represented different haplotypes. The blue and red histograms represented the father’s haplotypes. The orange and green histograms represented the mother’s haplotypes. In the embryos, the gray column represented the haplotype that was inherited from the normal parent. And in the carrier’s family number, the gray column represented the haplotype that wasn’t passed on to the carrier

In this study, two balanced translocation families were collected. In family 1, the carrier’s parents were normal, therefore their unbalanced embryos were used as reference. In family 2, the translocation was inherited from his mother, therefore paternal grandparent as reference. With our method BasePhasing, we obtained molecular karyotypes from all the 18 biopsied blastocysts. Of the 18 diagnosed blastocysts, 8 were unbalanced, 10 were balanced or normal (such rate might be a little higher than the published data due to the small sample size). BasePhasing analysis was performed in the 10 blastocysts, which verified that 5 were balanced carriers and 5 were normal embryos. Sample information and BasePhasing results of the two families are listed in Table 3. Specifically, the results of BasePhasing and PGH were exactly identical (Fig. 3). Such as in family 2, both of the two methods indicated embryo-2, embryo-3 and embryo-5 were translocation carrier embryos and embryo-1 and embryo-4 were structurally normal embryos.

**Table 3:** The characteristics and BasePhasing results of the patients in this study

Sample	Karyotype or Grade of blastocysts	ASA call rate	Karyomap-12 call rate	Results of BasePhasing	Results of Karyomap-12
Family 1					
Mother	46,XX	0.9862835	0.9842981		
Father	46,XY,t(1:5)(q21;q35) de novo	0.9897337	0.9838287		
Embryo-1	4BB	0.9643379	0.9607865	Normal	Normal
Embryo-2	4AB	0.9549406	0.9446636	Normal	Normal
Embryo-3	5BB	0.9460028	0.9430658	Carrier	Carrier
Embryo-4	5AB	0.9454245	0.9396804	Unbalanced	Unbalanced
Embryo-5	5AB	0.9342148	0.9312841	Normal	Normal
Embryo-6	5 BC	0.9626743	0.9603586	Carrier	Carrier
Embryo-7	4BB	0.9572856	0.951569	Unbalanced	Unbalanced
Embryo-8	4 BC	0.9062205	0.8713027	Unbalanced	Unbalanced
Family 2					
Mother	46,XX	0.9946477	0.9843602		
Father	46,XY,t(6;7)(q23;q34) mat	0.9950734	0.9892606		
Embryo-1	5BB	0.9273288	0.9067822	Normal	Normal
Embryo-2	5AB	0.9126197	0.8760443	Carrier	Carrier
Embryo-3	5AB	0.9505755	0.9167728	Carrier	Carrier
Embryo-4	5BB	0.9222872	0.9001667	Normal	Normal
Embryo-5	5BB	0.9429535	0.9313635	Carrier	Carrier
Embryo-6	5 AC	0.9406152	0.9309729	Unbalanced	Unbalanced
Embryo-7	5 BC	0.9419208	0.9253105	Unbalanced	Unbalanced
Embryo-8	5BB	0.9403009	0.9343323	Unbalanced	Unbalanced
Embryo-9	5 BC	0.9533952	0.945755	Unbalanced	Unbalanced
Embryo-10	5 BC	0.9147238	0.9127144	Unbalanced	Unbalanced

Mat: the balanced translocation was inherited from carrier’s mother. The karyotypes were identified by peripheral blood cells

In the meantime, the number of informative SNP loci within ±2Mbp regions flanking the breakpoints and the whole related chromosome was calculated (Table 4). It was clearly shown that there were more informative SNPs in the ASA bead chip than Karyomap-12 both in the related whole chromosomes and breakpoints flanking regions. For example, in chromosome 5 (0–180,915,260), there were 3774 SNPs available on the ASA chip, and the average number of informative SNPs per window was 83.4 (ranging from 13 to 151). While there were only 2732 SNPs available on the Karyomap-12 chip, and the average number of key SNPs per window was 60.4 (ranging from 6 to 118).

**Table 4:** The number of informative SNPs within ±2Mbp region flanking the breakpoint

Family	The location of breakpoint	Informative SNPs No. of the whole chromosome involved in translocation		Informative SNPs No. within ±2Mbp region flanking breakpoint	
		ASA/BasePhasing	Karyomap-12	ASA/BasePhasing	Karyomap-12
Family-1	chr1:151000000	4291	3104	27	23
	chr5:162000000	3774	2732	72	51
Family-2	chr6:144000000	2152	1377	61	46
	chr7:153000000	1659	1083	71	36

**Fig. 4:**
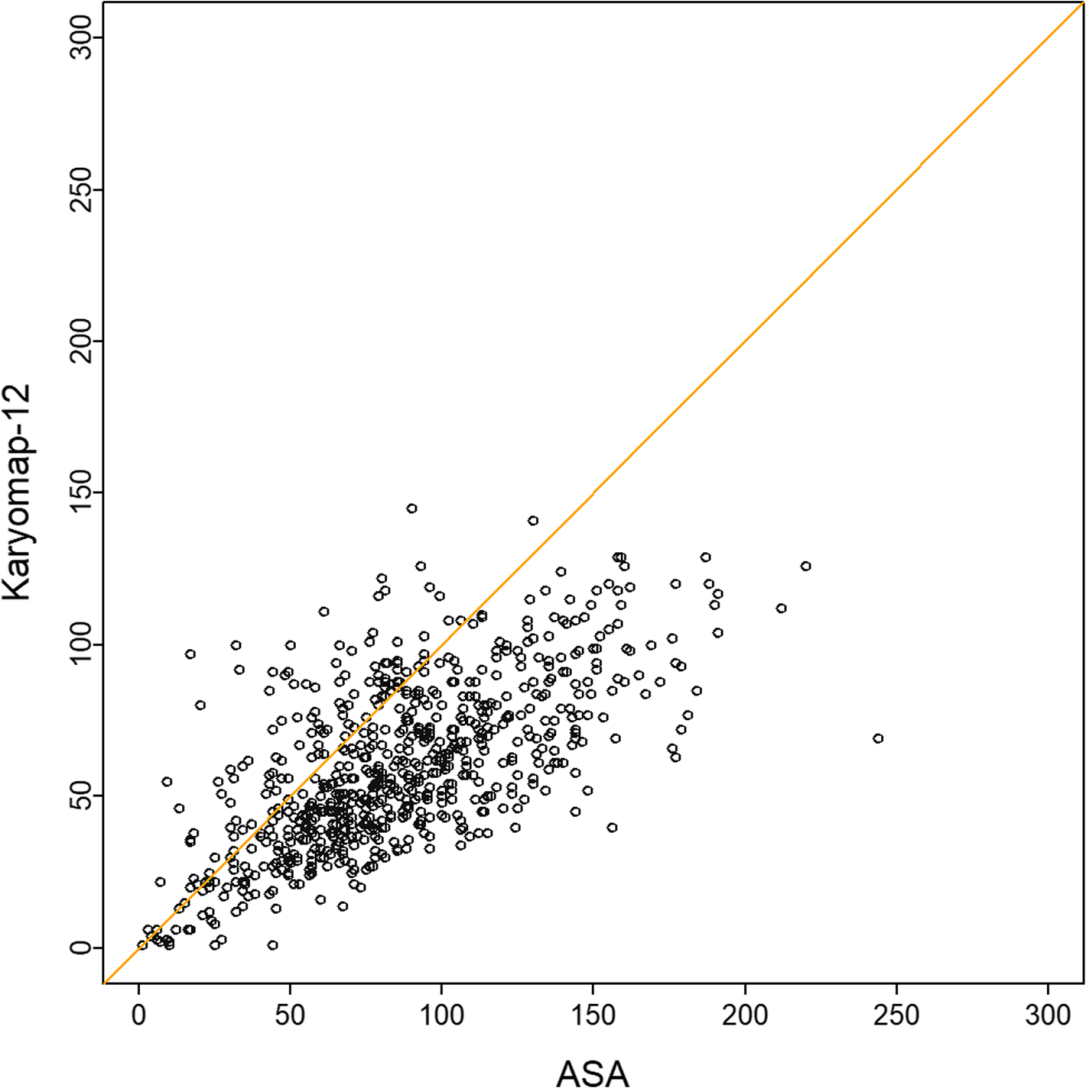
The accordance of the informative SNPs number between ASA and Karyomap-12 in family 2. The dots represent the windows in the whole genome; x axis indicated the number of informative SNPs in each window on ASA; and y axis indicated the number of informative SNPs in each window on Karyomap-12

We also calculated the number of informative SNP loci of family 2 in the whole human genome. The average number of informative SNPs was 85.9 in ASA bead chip and 59.4 in Karyomap-12 bead chip within ±2Mbp region along. The number of informative SNPs on ASA bead chip was a little bit more (Fig. 4). Based on this property of ASA bead chip, we asserted BasePhasing can perform better in balanced translocation carriers’ diagnosis.

After completing the BasePhasing analysis, embryo E1 in family 1 and embryo E4 in family 2 were transferred back to patients. For the two women that were pregnant after embryo transfer, cytogenetic analysis of amniotic fluid was required to be performed in the second trimesters. It was confirmed that the results predicted by BasePhasing and cytogenetic analysis of amniotic fluid cells were totally consistent.

## Discussion

Theoretically, only two kinds of gametes from alternate segregation, one with a normal karyotype and another with a balanced karyotype, can produce a viable conceptus in balanced translocation carriers, the remaining unbalanced gametes from other segregation patterns may lead to repeated miscarriage, infertility or newborns with congenital malformations [23–25]. Therefore, balanced translocation carriers are generally suggested to get a successful pregnancy with PGT. In 2016, Treff et al. [26] reported a new method using unbalanced embryos as a reference to distinguish between balanced translocation and normal blastocysts based upon SNP genotype. Recently Zhang et al. [21] and Wang et al. [22] utilized PGH to successfully distinguish between balanced and normal embryos prior to implantation from balanced translocation carriers accurately. Any SNP marker-based data can be applied for PGH analysis only if there are sufficient informative SNPs flanking the breakpoint to establish haplotype and program family linkage analysis. Meanwhile, the comprehensive chromosome screening (CCS) can also be completed with SNP allele frequency analysis in the single test.

In this study, we applied the ASA bead chip in BasePhasing pipeline and got accurate PGT results. By comparing the number of SNP loci in different MAFs and in 2Mbp continuous windows of ASA chip and karyomap-12 chip, it was verified that ASA could be used to perform haplotype linkage analysis of the whole genome. The average number of SNP loci in each window of ASA was 473.2, and up to 98.85% regions had more than 50 SNPs. Informative SNPs was 85.9 in ASA and 59.4 in Karyomap-12 bead chip within ±2Mbp region along of family 2. Through the analysis of whole gene amplification products of 3–10 cells of GM16457 cell line, we verified that ASA chip could be used for CNV testing of PGT, even a 5.3Mbp deletion was positive.

Compared with previous studies, several advantages of this research could be concluded. First, large numbers of SNPs could be used to perform the chromosome aneuploidies and haplotype linkage analysis, guaranteeing the accuracy of results. Second, without the need of precise translocation breakpoint location and personalized design, our method was universal for any kind of translocation. Third, the bead chip experiment was relatively simple and data analysis was convenient, which was suitable for clinical work. In addition, ASA based BasePhasing had over twice loci while only took about one quarter (22.5%) cost of Kayomap-12 bead chip (Additional file 2: Table S2). So BasePhasing would be one of the most suitable methods for PGT of balanced translocation carriers in clinical application at present. Nonetheless, for performing the analysis, one carrier’s family member or an unbalanced embryo should be used as a reference. Therefore, one limitation of our research was that the method didn’t apply to these patients both with de novo translocation and without an unbalanced embryo. To our knowledge, no current methods could effectively overcome this difficulty.

## Conclusions

Infinium ASA bead chip based BasePhasing pipeline shows good performance in PGT of balanced translocation. With the characteristics of simple operation procedure and accurate results, we demonstrate that BasePhasing is one of the most suitable methods to distinguish between balanced and structurally normal chromosome embryos from translocation carriers at present. In the meanwhile, we show a referable strategy to effectively expand the newly developed bead chips to clinical application. However, the sensitivity and specificity of BasePhasing should be further validated in a larger sample size. Furthermore, whether BasePhasing could be used to detect other single-gene diseases or other genetic diseases is worth further verifying.

## Additional files

Additional file 1: **Table S1.** Comparison of ASA and Karyomap-12 SNPs numbers at different minor allele frequency. (DOCX 17 kb)

Additional file 2: **Table S2.** The low cost of BasePhasing in PGT. (DOCX 16 kb)

## Abbreviations

ART: assisted reproductive technology
ASA: Illumina Infinium Asian Screening Array-24 v1.0
CCS: comprehensive chromosome screening
CNV: Copy number variation
DGV: Database of Genomic Variants
FISH: Fluorescent in-situ hybridization
GSA: Infinium Global Screening Array-24
IVF: In vitro fertilization
MAF: minor allele frequency
MDA: Multiple displacement amplification
PGD: Preimplantation genetic diagnosis
PGH: Preimplantation genetic haplotyping
PGT: preimplantation genetic testing
SNP: Single nucleotide polymorphism
WGA: Whole genome amplification
