# Supplementary material for: BasePhasing: a highly efficient approach for preimplantation genetic haplotyping in clinical application of balanced translocation carriers
Source: BMC Med Genomics. 2019 Mar 18;12:52. doi: 10.1186/s12920-019-0495-6 (PMC6423798; doi:10.1186/s12920-019-0495-6)
Supplement: Supplementary file 1 — Table S1. Comparison of ASA and Karyomap-12 SNPs numbers at different minor allele frequency. (DOCX 17 kb) [file 12920_2019_495_MOESM1_ESM.docx]

**Table S1 Comparison of ASA and Karyomap-12 SNPs numbers at different minor allele frequency**

| MAF^a^ | Karyomap-12 | | ASA | |
| --- | --- | --- | --- | --- |
|  | SNPs No. | rate | SNPs No. | rate |
| 0-0.05 | 39337 | 13.35% | 342080 | 48.97% |
| 0.05-0.10 | 27326 | 9.28% | 65242 | 9.34% |
| 0.10-0.15 | 32072 | 10.89% | 53918 | 7.72% |
| 0.15-0.20 | 32073 | 10.89% | 46622 | 6.67% |
| 0.20-0.25 | 30614 | 10.39% | 41315 | 5.91% |
| 0.25-0.30 | 28530 | 9.68% | 35520 | 5.09% |
| 0.30-0.35 | 27552 | 9.35% | 31541 | 4.52% |
| 0.35-0.40 | 26145 | 8.87% | 28893 | 4.14% |
| 0.40-0.45 | 25747 | 8.74% | 27206 | 3.89% |
| 0.45-0.50 | 25206 | 8.56% | 26163 | 3.75% |
| Total | 294602 | 100.00% | 698500 | 100.00% |

^a^minor allele frequency.
