# Supplementary material for: BasePhasing: a highly efficient approach for preimplantation genetic haplotyping in clinical application of balanced translocation carriers
Source: BMC Med Genomics. 2019 Mar 18;12:52. doi: 10.1186/s12920-019-0495-6 (PMC6423798; doi:10.1186/s12920-019-0495-6)
Supplement: Supplementary file 2 — Table S2. The low cost of BasePhasing in PGT. (DOCX 16 kb) [file 12920_2019_495_MOESM2_ESM.docx]

**Table S2 The low cost of BasePhasing in PGT**

| Bead Chip | Each Sample Cost | | | PGT Cost of One Family (3B+6E)^a^ |
| --- | --- | --- | --- | --- |
|  | Chip | WGA | gDNA extraction |  |
| Karyomap-12 array | $350 | $30 | $1.5 | $2284.5 |
| ASA/BasePhasing | $55 | $30 | $1.5 | $514.5 |

^a^we set one PGT family including 3 blood samples and 6 embryos.
